# Next generation sequencing of benzo(a)pyrene-induced *lacZ* mutants identifies a germ cell-specific mutation spectrum

**DOI:** 10.1038/srep36743

**Published:** 2016-11-10

**Authors:** Jason M. O’Brien, Marc A. Beal, Carole L. Yauk, Francesco Marchetti

**Affiliations:** 1 Environmental Health Science and Research Bureau, Health Canada, Ottawa, ON, K1A 0K9, Canada

## Abstract

*De novo* mutations are implicated in a variety of genetic diseases and arise primarily in the male germline. We investigated whether male germ cells have unique mechanisms for spontaneous or chemically-induced mutation relative to somatic cells using the MutaMouse model. We recovered *lacZ* transgenes from sperm 42 days after a 28-day exposure to benzo(a)pyrene (BaP, 100 mg/kg/day) to assess mutations arising in dividing spermatogonia. BaP caused a 3.4-fold increase in *lacZ* mutant frequency over controls which increased to 4.1-fold after clonal correction. We then used next generation sequencing to compare the spontaneous and BaP-induced mutation spectra in sperm and bone marrow. The spontaneous spectrum in sperm had significantly more G:C to A:T transitions and fewer mutations at A:T basepairs than bone marrow. BaP predominantly induced G:C to T:A transversions in both cell types, and both were enriched for mutations at CpG dinucleotides. However, BaP induced significantly more deletions in sperm, but more G:C to A:T transitions and G:C to C:G transversions in bone marrow. Differences in error-prone translesion DNA synthesis polymerases may underlie the observed spectrum differences between sperm and bone marrow. These findings suggest that mutations in sperm can arise via mechanisms that are unique to male germ cells.

Over long periods of time, germline mutation is one of the primary driving forces of evolution. However, short term increases in mutation rates can lead to unwanted increases in the incidence of genetic disease and cancer. Recent studies using next generation sequencing confirm that the male germline is the primary source of *de novo* mutations (i.e., genetic alterations in an offspring that are not present in the parents)^1^,^2^,^3^,^4^. These efforts have focussed primarily on the rate of male-mediated inherited mutations, but few have explored what environmental factors are responsible for differences among males. Thus, environmental variables that contribute to *de novo* mutations remain poorly characterized.

Elevated mutagenicity in the male germline is believed to be due to the continuous replication of spermatogonial stem cells^1^. Indeed, the number of mutations inherited by offspring is proportional to paternal age at the time of conception, supporting the hypothesis that the male germline mutation rate is directly correlated with the number of spermatogonial cell divisions^3^,^4^. In addition, there are several mechanistic factors, some of which are unique to males, that may contribute to germline specific mutation^5^: (a) male germ cells progressively lose DNA repair capacity as they advance through spermatogenesis^6^,^7^; (b) the chromatin remodeling required for the replacement of histone proteins with protamines during spermiogenesis^8^ may promote additional opportunities for misrepair of endogenous strand breaks^9^; (c) the process of meiosis provides opportunities to affect nucleotide composition that are unique to germ cells; and, (d) post-meiotic haploid germ cells cannot rely on sister chromatids as templates for homology-based repair mechanisms^10^. Thus, there are multiple mechanisms unique to male germ cells by which mutations can arise. In addition to influencing mutation rate, these factors may also contribute to the generation of different types of mutation (ie, mutation spectrum) in male germ cells relative to other cell types. It stands to reason, then, that mutagenic chemicals may induce mutations via germ cell-specific mechanisms, resulting in a unique spectrum of mutations relative to other cell types. However, this has never been clearly demonstrated.

The transgenic rodent (TGR) mutation assay is an internationally supported method for testing the *in vivo* mutagenicity of chemicals^11^. The assay uses rodents that harbor mutation-reporting transgenes in their genomes. The transgenes are easily recovered, from virtually any tissue, and screened for induced mutations. TGR models offer a practical, fast and efficient means to assess the mutagenicity of chemicals in germ cells and compare the responses with somatic tissues^12^,^13^,^14^,^15^. One of the many benefits of the TGR mutation assay is that the recovered transgenes can be sequenced to provide additional data on the types of mutations induced, which can be used for developing and supporting mechanistic hypotheses. Previously, sequencing data were limited to very low copy numbers of the smaller *cII* transgene due to technical restrictions. However, when coupled with next generation sequencing (NGS) technologies, the TGR assay can be used to generate a high-resolution mutation spectrum of the larger *lacZ* transgene, providing coverage of a much greater number of mutants^16^,^17^. Thus, NGS-*lacZ* analysis allows the detection of subtle differences in mutation spectra that may otherwise be missed when analyzing a smaller sample of mutants.

Benzo(*a*)pyrene (BaP) is a ubiquitous environmental pollutant and Group 1 human carcinogen^18^ that causes both somatic and male germline mutations^12^,^13^,^19^,^20^. The mechanisms of BaP mutagenicity have been well characterized and reviewed for somatic tissues^18^,^21^. The primary mechanism is the faulty replication of unrepaired DNA adducts formed by a BaP metabolite, benzo(*a*)pyrene-7,8-dihydrodiol-9,10-epoxide (BPDE). BPDE adducts occur predominantly at the N^2^ position of guanosine, resulting in an increased frequency of G:C to T:A transversions^22^,^23^,^24^. Several other DNA-reactive by-products of BaP metabolism, such as BaP radical cations and reactive oxygen species (ROS), also cause varying degrees of other types of mutations. The BaP mutation spectrum is less well characterized in germ cells. While BaP can induce mutations during various phases of spermatogenesis, including stem cells, dividing spermatogonia appear to be the most sensitive^12^,^13^,^19^,^20^,^25^,^26^. The characteristic G:C to T:A transversion also appears to be the primary BaP-induced mutation detected in sperm^13^. However, a high resolution mutation spectrum has not yet been generated to assess the effect of BaP, or any other chemical, on all mutation types in male germ cells.

We hypothesize that mutations can arise via mechanisms that are unique to male germ cells. A testable prediction of this hypothesis is that somatic cells and male germ cells will yield distinct spectra of mutation types both before and after exposure to a chemical mutagen. Here, we use the TGR mutation assay coupled with NGS to produce high-resolution spectra of mutation types in the germ cells of untreated and BaP-exposed male mice. We then compare these to the spontaneous and BaP-induced mutation spectra in bone marrow^16^ and speculate on the possible mechanisms responsible for any spectral discrepancies between these tissues. This study provides important evidence for elucidating whether or not there are germ cell-specific mechanisms for spontaneous and BaP-induced mutations.

## Results

### LacZ mutant frequency

The animals used in the present study are a subset from a previously reported dose-response study on the induction of *lacZ* mutations in dividing spermatogonia^26^. MutaMouse males used in the present study were exposed to a vehicle control (olive oil, n = 5) or 100 mg/kg bw/day BaP (n = 6) by oral gavage for 28 consecutive days. We selected this dose due to its high mutagenicity in germ cells^26^, which allowed us to produce a high resolution spectrum of BaP-induced mutations. This dose is on the higher end of the dose-response curve, and is not necessarily representative of an environmentally relevant exposure. Thus, results should be interpreted for mechanistic considerations only and not as indicative of environmental risk. Spermatozoa from the cauda epididymis were collected 42-days after exposure to target cells that were dividing spermatogonia at the end of the exposure period^14^. A positive selection assay was used to recover DNA harboring mutated copies of the *lacZ* gene from the spermatozoa (in the form of viral plaques grown on a lawn of host bacteria). The *lacZ* mutant frequency (MF) in the BaP group (MF = 13.1 × 10^−5^) was 3.4-fold higher (p < 0.001) than in controls (MF = 3.9 × 10^−5^), as shown in Table 1.

### Sequencing and clonality

We used NGS to sequence a total of 1591 *lacZ* mutant plaques and 200 titre plaques (assumed wildtype) derived from spermatozoa from the cauda epididymis in five control (578 mutant plaques) and six BaP-treated animals (1013 mutant plaques). Sequence data are available on the NCBI Sequence Read Archive (http://www.ncbi.nlm.nih.gov/sra) under the BioProject accession number PRJNA342798. Sequencing generated 80, 327, 390 reads with an average read-length of 129 bp resulting in a total of 9.2 × 10^9^ high quality base calls (≥Q20). For the purpose of this study, sequencing depth is defined as the average number of base calls at any nucleotide position along *lacZ*. Alignment to the reference sequence resulted in an average sequencing depth of approximately 2700-fold (range = 1370-fold to 5124-fold) per identified copy of *lacZ* (i.e. each base in each mutant copy of *lacZ* that we identified was sequenced on average approximately 2700 times). A similar study found that a sequencing depth of 35-fold was sufficient for highly accurate mutation identification^17^. From this alignment, a total of 1117 mutants were identified (393 in controls and 724 in the BaP group) resulting in 70% recovery of the original input (1117 recovered mutants ÷ 1591 input mutants). No mutations were detected in the wildtype plaques. Mutations that occurred more than once per animal were considered to have originated from a single independent event via clonal expansion. Using the limit of detection/linear model^16^ to correct for clonal expansion we identified 427 independent mutations (131 in controls and 296 in the BaP-exposed group). As shown in Table 1, the average clonality in the germ cells of BaP-exposed animals (24.8%) was lower than in control animals (41%). This difference, however, was not statistically significant (p = 0.122, t-test), owing largely to the high variability in clonality among samples. The *lacZ* MFs were then corrected for the varying degrees of clonality (Table 1). Clonal correction increased the relative fold change in mutational response between controls and BaP-treated samples (fold change = 3.4 before; fold change = 4.1 after correction), and reduced the variability in the BaP group (standard deviation = +/−3.4 (26%) before; standard deviation = + /−1.0 (11%) after correction).

### BaP-induced mutation spectrum in germ cells

**Figure 1:**
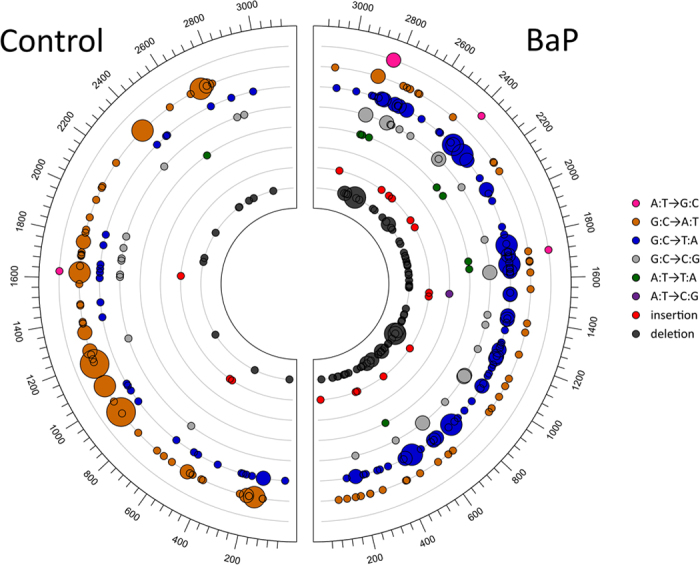
The type and position of all independent spontaneous (control) and benzo[a]pyrene(BaP)-induced *lacZ* mutations detected in sperm collected 42 days after the treatment period. Circle size is proportional to the number of times a mutation was detected in different animals.

**Figure 2:**
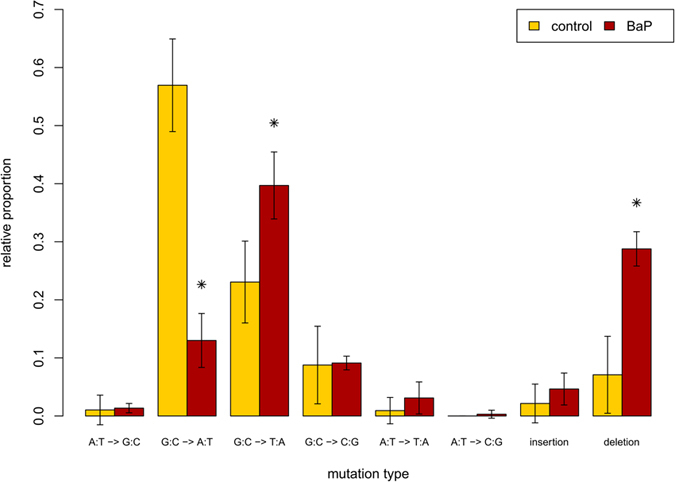
The proportion of all independent spontaneous (control) and benzo[a]pyrene (BaP)-induced *lacZ* mutations detected in sperm collected 42 days after the treatment period. *Indicates significant differences (p < 0.05) between control and BaP groups.

The type, position, and frequency of independent mutations in the control and BaP groups are shown graphically along the *lacZ* gene in Fig. 1 and described in detail in Supplementary Table 1. One large 899 bp deletion, which was confirmed by Sanger sequencing, was identified in one of the control animals. This large deletion was omitted from downstream mutation spectrum analysis. All other reported deletions are 1 or 2 bp in length. The *lacZ* mutation spectrum in the sperm of BaP-exposed animals showed significant differences (p = 0.01) with respect to the control group when compared using a Monte Carlo generated *X*^2^ distribution. The proportional spectra of mutation types for each treatment group are shown in Fig. 2. The most frequent types of mutation in the control group were G:C to A:T transitions (57%), followed by G:C to T:A transversions (22%). The proportional spectrum of mutation types in the BaP group was significantly different compared with the control group (p < 0.001; Fisher’s exact test). A multiple comparison of the individual mutation types showed that the most significant differences were the decreased proportion of G:C to A:T transitions (from 57% in controls to 13% in the BaP group), and the elevated proportion of G:C to T:A transversions (from 23% to 39%) and deletions (from 7% to 29%) in the BaP-induced mutation spectrum. When the spectra were corrected for differences in MF (by multiplying the proportion of mutation types by the MF of each group (Supplementary Figure 1), it was apparent that BaP exposure increased the frequency of all mutation types compared to background levels except for G:C to A:T transitions.

To analyze the influence of the sequence context on mutation formation, we compared the proportion of mutations that occurred at G:C or A:T base-pairs in each treatment group, and checked whether the nucleotide position of each mutation occurred within or adjacent to a nucleotide homopolymer repeat or at areas with one or more CpG dinucleotides (Supplementary Table 2, summarized in Table 2). These results show that, in addition to the overall increase in the proportion of G:C to T:A transversions, there was also a statistically significant increase (p < 0.05, Fisher’s exact test) in the proportion of mutations that occurred at A:T nucleotides, from 3.8% in the control group to 18.2% in the BaP group. We also detected a marginally significant (p < 0.1) increase in the proportion of all mutations that occurred at CpG dinucleotides in the BaP group. When divided into mutation types, it became apparent that the increased proportion of BaP-induced mutations at A:T nucleotides was predominantly due to deletions: 43% of the deletions that occurred in BaP-treated animals were at A:T nucleotides. However, this increased proportion was not significantly greater than the control group. The elevated proportion of mutations at CpG dinucleotides in the BaP group appeared to be mostly G:C to T:A and G:C to C:G transversions, which occurred significantly more frequently than in controls by approximately 30%. The occurrence of homopolymers did not have an obvious impact on the different types of mutations across treatment groups.

Several positions along the *lacZ* gene were mutated in more than one animal (Fig. 1, Supplementary Table 3) indicating possible hotspots. A position was considered a hotspot if a mutation occurred there in more than one animal per dose-group. Eighteen hotspots were identified in mice from the control group. Nearly half (43%) of the mutations in the control group occurred at these hotspots. All of the hotspots identified in the control group occurred at G:C basepairs, at which the majority of mutations were G:C to A:T transitions. None of the control hotspots had deletions or insertions. In the BaP treatment group, we identified 48 hotspots. As was observed in control mice, 43% of all independent mutations identified in the BaP-treated animals occurred at these hotspots. Most of the hotspots in the exposed group were also at G:C basepairs; however, around 11% of the hotspots occurred at A:T basepairs. Approximately 56% of the mutations in hotspots of BaP-exposed mice were G:C to T:A transversions, and 21% were deletions, most of which occurred at G:C basepairs. Between both groups, a total of 63 unique hotspots were identified. Six of these had mutations only in the control group, 36 had mutation only in the BaP group, and 21 of the identified hotspots had mutations in both control and BaP-exposed groups. Finally, we determined the codon and amino acid changes caused by each mutation (Supplementary Table 4). Similar proportions of premature stop codons (56.4% vs 48.2%) and missense mutations (43.6% vs 46.2%) were observed in control and BaP-treated mice, respectively. No silent mutations were observed in controls, while they represented 5.6% of the mutations induced by BaP (but this was not significantly higher than controls). These silent mutations likely represent “hitch-hiker” mutations that co-occurred with other mutations that were detectable by the positive selection assay^16^.

### Comparison of sperm and bone marrow mutation spectra

**Figure 3:**
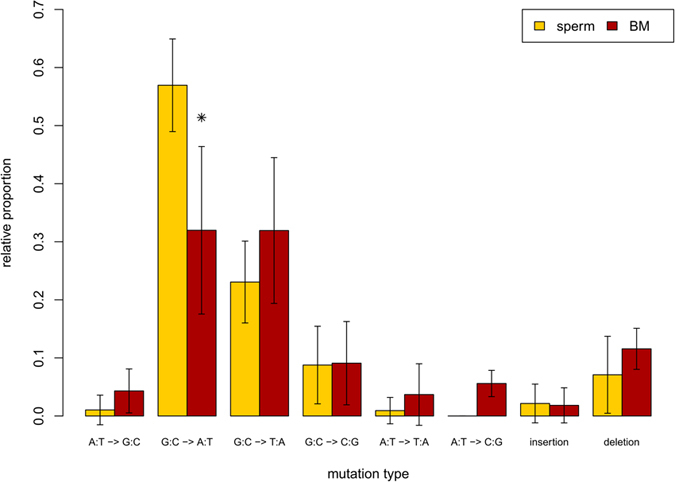
Comparison of the proportion of spontaneous mutation types in the *lacZ* gene collected from sperm and bone marrow. *Indicates significant differences (p < 0.05) between tissues. Bone marrow data are from a previously published study^16^.

**Figure 4:**
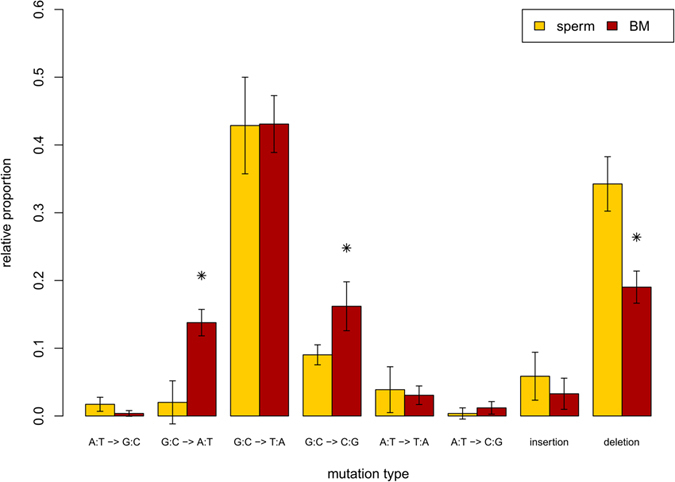
Comparison of the proportion of benzo[a]pyrene (BaP)-induced mutation types in the *lacZ* gene isolated from sperm and bone marrow. *Indicates significant differences (p < 0.05) between tissues. Bone marrow data are from a previously published study^16^.

We compared the control and BaP-induced mutation spectra in sperm to the control and induced spectra in bone marrow published previously^16^ (Fig. 3 and Table 3). The background mutation spectrum in sperm was largely similar to the background spectrum in bone marrow (Fig. 3). However, there were two key differences: sperm had significantly (p < 0.001) more G:C to A:T transitions (57% in sperm vs 32% in bone marrow), and significantly (p < 0.001) less mutations at A:T basepairs (3.8% in sperm vs 18.8% in bone marrow) (Table 3). Because the magnitude of the BaP-induced response was much greater in bone marrow (MF = 701.7 × 10^−5^ in bone marrow; MF = 13.1 × 10^−5^ in sperm), BaP-induced mutation spectra in sperm and bone marrow were corrected for differences in background mutation spectrum and for differences in the BaP-induced mutation frequency (Fig. 4 and Table 3). G:C to T:A transversions were the most common mutation following BaP exposure in both tissues, and were induced at nearly identical proportions in both cell types. BaP induced a significantly greater proportion of G:C to A:T transitions and G:C to C:G transversions in bone marrow, but a greater proportion of deletions in sperm. Thus, the mutation spectra in sperm and bone marrow show differences before and after BaP exposure.

## Discussion

We used the TGR mutation assay to recover mutated *lacZ* genes from spermatozoa from the cauda epididymis of untreated and BaP-exposed male mice. Using NGS we generated the most detailed spontaneous and BaP-induced mutation spectrum in sperm to date (Figs 1 and 2), which provided insights into the mechanistic underpinnings of the spontaneous and induced mutations in male germ cells. We detected the characteristic BaP signature of increased G:C to T:A transversions in sperm, but also identified several significant differences in the mutation spectrum of sperm compared to the bone marrow of similarly treated animals (Figs 3 and 4). These results suggest that the mutagenic mechanisms operating in germ cells may differ from those in somatic tissues.

One of the major benefits of sequencing mutant genes recovered from the TGR assay is the ability to correct for clonal expansion^16^ that can artificially inflate the observed MF. In the present study, clonality was lower in the BaP-treatment group (24.8%) compared with controls (41%). Correcting for clonality had the effect of increasing the fold-change of the mutational effect and reduced the relative inter-individual variability within the BaP group (Table 1). Thus, clonal correction improved the TGR assay’s ability to detect statistically significant effects.

Using NGS we sequenced ~5-fold more mutants than the combined number of mutants sequenced from all previous studies that have recovered transgenes (*cII* or *lacZ*) from germ cells^13^,^15^,^27^. This allowed us to resolve high resolution mutation spectra and identify treatment- and tissue-specific effects. However, it is possible that an even greater number of animals or sequenced mutants would have allowed us to resolve more subtle differences that were not detectable with our sample size, especially for mutation types with lower frequency such as those occurring at A:T base pairs.

We found that spontaneous mutations occurred almost entirely at G:C basepairs (96.2%, Table 2), which is significantly greater than what would be expected by chance (p < 0.001, Fisher’s Exact) given that the entire *lacZ* sequence consists of 57% G:C base-pairs. Consistent with previous reports using traditional Sanger sequencing^15^, the predominant forms of spontaneous mutations were G:C to A:T transitions followed by G:C to T:A transversions. The bias for spontaneous G:C to A:T transitions is believed to arise primarily from the deamination of methylated cytosine^28^. However, this mechanism does not explain the G:C to A:T bias in non-CpG regions^29^, nor does it explain the overall G:C to A:T bias in species with little to no methylation^30^. In the present study, 56% (985/1754) of all G:C base-pairs in the *lacZ* sequence are in CpG dinucleotides, but only 49% (37/75) of the spontaneous G:C transition mutations occurred at CpGs, which is not significantly different from chance (p = 0.287, Fisher’s exact). This is not consistent with cytosine deamination as the primary mechanism for spontaneous G:C transition mutations, given that all of the CpGs of the *lacZ* transgene are known to be heavily methylated in all tissues, including testes^31^,^32^. A similar lack of selection for CpG sites was also observed in the *lacI* and *cII* transgenes of Big Blue rats and mice^33^, leading the authors to conclude that mechanisms other than spontaneous deamination of methylcytosine were driving spontaneous mutation.

Other proposed mechanisms for the G:C to A:T spontaneous mutation bias include DNA damage from ROS^34^, or G:C tautomerization caused by proton transfer across the base-pair hydrogen bonds^29^,^35^. In the present study, there was a marginally significant enrichment (p = 0.066) in the proportion of G:C to A:T transitions that occurred at dipyrimidine sites. In fact, 81% (61/75) of spontaneous transitions occurred at dipyrimidine sites, whereas only 71% (1235/1737) of all G:Cs in the *lacZ* gene are in a dipyrimidine context. These results suggest that the occurrence of dipyrimidines may be an important factor in the observed spontaneous mutation spectrum, especially for spontaneous G:C to A:T transitions.

Spontaneous G:C to T:A transversions are likely associated with damage from ROS produced by aerobic metabolism^28^. One of the most common forms of ROS damage is the oxidation of guanosine into 8-oxo-7-hydrodeoxyguanosine (8-oxo-dG), which mispairs with adenosine causing high levels of spontaneous G:C to T:A transversions. As 8-oxo-dG is readily detectable in the testes and sperm of mice^36^,^37^,^38^, we speculate that this mechanism is responsible for the high proportion of G:C to T:A transversions in the spontaneous mutation spectrum in sperm.

The spectrum of spontaneous mutations in sperm was comparable to that observed in bone marrow (Fig. 3). As in sperm, CpG frequency did not influence the occurrence of G:C to A:T transitions. Also as in sperm, 80% (36/45) of the G:C to A:T mutations in bone marrow occurred at dipyrimidines. However, this observation was not statistically significant for bone marrow due to the few number of G:C to A:T transitions recovered from this tissue (p = 0.24). In fact, germ cells had a significantly higher proportion of G:C to A:T transitions (p < 0.001) than bone marrow, suggesting that a dipyrimidine-based mechanism of spontaneous mutation may be more predominant in sperm. All other mutation types in the spontaneous mutation spectra, including G:C to T:A transversions, had similar proportions (Fig. 3) and sequence context (Tables 2 and 3) among the two cell types. Overall, these results show that subtle differences exist in the spontaneous mutations that occur in sperm versus bone marrow.

BaP’s mutagenic mode of action in germ cells is assumed to be similar to somatic tissues. BaP is metabolized by cytochrome P450s and epoxide hydrolase to form the predominant mutagenic metabolite BPDE, which forms DNA adducts on N2 of guanosine causing G:C to T:A transversions during replication^39^,^40^,^41^. In the present study, the BaP-induced mutation spectrum in sperm was dominated by G:C to T:A transversions (Figs 1 and 2), occurring primarily at CpG dinucleotides (Table 2). This is consistent with a BPDE mode of action, since methylated CpGs are the preferential target for BPDE adduct formation^42^,^43^. Similarly, Olsen *et al*.^13^ reported an increased proportion of G:C to T:A transversions after sequencing ~50 mutant *cII* genes from spermatozoa of BaP-treated mice. BaP also induces mutations via several other minor mechanisms. BPDE forms adducts at N6 of adenosine and N4 of cytosine, but at a lower frequency than with guanosine. BaP metabolism also results in the production of ROS species that can form various types of DNA damage, including 8-oxo-dG^44^, and other secondary adducts that can contribute to the mutagenic profile of BaP. These various mechanisms are likely to be at least partially responsible for the more modest increase in other mutation types.

The BaP-induced mutation spectrum in germ cells and bone marrow shared many characteristics (Fig. 4). The predominant mutation type in both cell types was the G:C to T:A transversion, and both spectra were enriched for mutations at CpG dinucleotides (Tables 2 and 3), which is the typical BaP mutation signature. These results suggest that the major mechanism for BaP-induced mutation in both sperm and bone marrow is via BPDE adducts on guanosine bases. However, there were also some notable differences: sperm from BaP-exposed mice had proportionally fewer G:C to A:T transitions and G:C to C:G transversions than bone marrow, and a greater proportion of deletions. Small insertions and deletions (indels) are most often associated with replication errors due to polymerase slippage across homopolymeric sequences with BPDE-DNA adducts. In fact, all identified deletion hotspots occurred at homopolymeric regions, indicating they are important for BaP-induced deletions. However, only ~25% and 22% of BaP-induced deletions were associated with homopolymers, in sperm and bone marrow respectively, indicating that homopolymer slippage is not the primary mechanism responsible for the preponderance of deletions in sperm. We suggest that error-prone translesion DNA synthesis polymerase polymerase kappa (polK), and perhaps other Y-family polymerases^45^,^46^,^47^, are likely involved. These polymerases have large active sites that can accommodate bulky lesions on the template strand, thus permitting synthesis through damaged regions. Due to their tolerant active sites, strand slippage and erroneous base-pairing are more frequent compared to normal polymerases and occur independent of the presence of homopolymer repeats^48^. The Y-family polymerases are involved in the repair of BaP adducts^49^ and have a mechanism that would result in elevated deletions in non-polymer regions, as well as increased rates of all possible base substitutions. Further, their elevated expression in the testes^45^,^50^,^51^ may explain the greater proportion of homopolymer-independent deletions in sperm observed in the present study.

In conclusion, analysis of high-resolution mutational spectra by NGS showed that there is overlap in BaP’s mutagenic mode of action between germ cells and bone marrow, although some notable differences were observed. Differential distribution of BaP metabolites between tissues^52^ cannot be discounted as a mechanism contributing to the observed differences in the mutation spectra between germ cells and somatic cells. However, our results point to potential differences in DNA repair mechanisms operating on BaP-induced damage in the two cell types. Our results are in accordance with the testable prediction that male germ cells will have a divergent mutation spectra in untreated and mutagen-exposed animals compared with somatic tissues. Thus, these findings support the hypothesis that mutations in sperm can arise via mutational mechanisms that are unique to male germ cells.

## Materials and Methods

### Animal exposures and tissue collection

All protocols involving animal use were approved by the Health Canada Ottawa Animal Care Committee and animal manipulations were carried out in accordance with the guidelines and regulations of the Canadian Council on Animal Care. Experiments were conducted following the recommendations in OECD guideline TG488^11^. Briefly, MutaMouse males (8–10 weeks old) were orally exposed to an olive oil vehicle control (n = 6) or 100 mg/kg bw BaP (n = 5) dissolved in olive oil for 28 consecutive days via gavage. Mice were euthanized by cervical dislocation under isofluorane anaesthesia 42 days after the end of the exposure period. Cauda epididymides were collected, flash frozen in liquid nitrogen, and stored at −80 °C. Sperm were later collected from the cauda as described previously^14^.

### LacZ Mutation Assay

Genomic DNA was isolated from spermatozoa from the cauda epididymis by digestion with proteinase K and β-mercaptoethanol, followed by phenol/chloroform extraction, and ethanol precipitation as described previously^14^. The *lacZ* MF was determined in genomic DNA as previously described^14^. Briefly, viral transgene vectors were recovered from ~1–4 μg of DNA using Transpack Packaging Extract kits (Agilent Technologies, Mississauga, ON, Canada) according to the manufacturer’s instructions. *E. Coli (lacZ*^−^/*galE*^−^) were infected with the recovered viral vectors and grown on agar containing 0.3% phenyl-β-D-galactopyranoside (P-Gal) to detect mutants, or on agar without P-Gal to determine the total number of plaque-forming units (pfu). A minimum of 125,000 total pfu were scored for each animal. MF was calculated as the number of mutant plaques divided by the total pfu count. Statistical differences in MF between treatment groups were determined by logistic regression using the glm function in R^53^ with a quasibinomial error distribution to account for overdispersion of the data.

### Mutant Sequencing

Mutant plaques recovered from spermatozoa from the cauda epididymis were collected for sequencing. Sequencing was performed using an Ion Proton sequencer (Life Technologies, Carlsbad, CA) as described previously^16^. Briefly, for each animal, mutant plaques were pooled into a microcentrifuge tube containing sterile water (average of 144 plaques per animal). Tubes were boiled to melt the agar and an aliquot of the suspension was used as template for a 30-cycle PCR amplification of the *lacZ* gene. To control for PCR errors, each sample was amplified and sequenced in duplicate. The PCR products were purified using QIAquick PCR purification kit (Qiagen). Libraries were prepared from each purified PCR sample using Ion Xpress Plus kits and an AB Library Builder (Ion Xpress Plus Library Protocol v. 1.00) following the manufacturer’s directions with the following exceptions: we used a custom shearing time of 10 minutes and replaced the Agencourt AMPure XP Reagent with SPRIselect Reagent (Beckman Coulter, Brea, CA). The ends of each library were ligated with P1 adapters and a unique barcode A adapter from the Xpress barcode adapter kit. Fragments between ~175–225 bp were selected from each library using an E-Gel Safe Imager, purified using the Invitrogen PureLink Quick Gel Extraction kit, and amplified using the Library Amplification Primer Mix (Ion Plus Fragment Library Adapters kit) and High Fidelity Platinum PCR SuperMix. Barcoded libraries were pooled together to a final total concentration of 8 pM. Pooled libraries were amplified by emulsion PCR using Ion Sphere particles on an Ion OneTouch2 system. Particles containing PCR products were enriched using an Ion OneTouch ES. Enriched particles were loaded onto an Ion P1 chip (version 1) and sequenced.

Bioinformatic analysis of sequence data was conducted as described previously^16^. Briefly, raw sequence data were interpreted using a Proton Torrent Server (version 3.6.2). Reads were trimmed and aligned to the reference *lacZ* sequence (GenBank ID: J01636.1) using Bowtie2^54^. Alignment pileup was performed using SAMtools^55^. The proportion of reads containing base-substitutions, insertions, or deletions (indels) relative to the reference sequence was determined from the pileup, adjusting for the false mutation proportion (which estimates PCR and sequencing errors)^16^. Indels with a false mutation proportion higher than the highest false mutation proportion for base substitution calls were ignored to reduce false indel calls that result from homopolymer sequencing errors. The mutation-calling-threshold was conservatively set based on the number of plaques sequenced for each animal as follows: mutation-calling-threshold = 1/#plaques sequenced for that animal (e.g., if 100 plaques were sequenced, the required threshold would be at least 1 read with the mutation/100 plaques sequenced = 0.01). Deviations from the reference sequence were called as mutations (corrected for false mutation proportion) if they were above the mutation-calling-threshold in both technical replicates. Mutations that occurred more than once per animal were considered to originate by clonal expansion from a single independent event. The number of clones for each mutation per animal was determined by dividing the average corrected proportion of deviant reads between technical replicates by the mutation-calling-threshold. This value was then adjusted using a limit of detection/linear model to account for the imprecise quantification of clonality near the limit of detection, as described previously^16^.

Only the independent mutations were used to determine the mutation spectra for each treatment group. Significant differences in mutation spectra, when considering both nucleotide position and mutation type, were determined using a Monte Carlo generated *X*^2^ distribution^56^. Significant differences in mutation spectra when considering only mutation type were determined using a Fisher’s exact test. To determine which specific types of mutations were significantly different between the control and treatment groups, a Fisher’s exact test was performed on 2 × 2 sub-tables for each mutation type, followed by a Bonferroni correction for multiple comparisons. Each sub-table comprised a count of the mutation type of interest and a pooled count of all other mutation types in each treatment groups.

## Additional Information

**How to cite this article**: O’Brien, J. M. *et al*. Next generation sequencing of benzo(a)pyrene-induced *lacZ* mutants identifies a germ cell-specific mutation spectrum. *Sci. Rep*. **6**, 36743; doi: 10.1038/srep36743 (2016).

**Publisher’s note**: Springer Nature remains neutral with regard to jurisdictional claims in published maps and institutional affiliations.

## Supplementary Material

Supplementary Information

Supplementary Table S1

Supplementary Table S2

Supplementary Table S3

## Figures and Tables

**Table 1:** *LacZ* mutant frequency in sperm collected 42 days after a 28-day exposure to benzo(a)pyrene, and clonal correction.

Dose (mg/kg bw/day)	Animal ID	Raw data				Clonality		Clonal correction			
		MF × 10^−5^	Average MF × 10^−5^	SD	Fold change	% clonality	Average % clonality	Corrected MF × 10^−5^	Average MF × 10^−5^	SD	Fold change
0	1	1.6	3.9	1.8 (46.2%)	—	56.8	41.0	0.7	2.3	1.2 (52.2%)	—
	2	4.6				14.3		3.9			
	3	4.7				58.5		2.0			
	4	2.5				40.6		1.5			
	5	3.3				26.8		2.4			
	6	6.7				49		3.4			
100	7	13.4	13.1	3.4 (26.0%)	3.4	27.8	24.8	9.7	9.5	1.0 (10.5%)	4.1
	8	13.3				35.2		8.6			
	9	11.2				17.2		9.3			
	10	9.2				4.8		8.8			
	11	18.4				39.1		11.2			

MF: mutant frequency.

SD: standard deviation.

**Table 2:** Analysis of sequence context of *lacZ* mutations identified in spermatozoa from the cauda epididymis of male mice exposed to benzo(a)pyrene.

	ALL MUTATIONS		Insertions		Deletions	
	Control	BaP	Control	BaP	Control	BaP
#Mutations	131	296	3	13	11	86
A:T	5 (3.8%)	**54 (18.2%)**	1 (33.3%)	3 (23.1%)	2 (18.2%)	37 (43%)
G:C	125 (96.2%)	**242 (81.8%)**	2 (66.7%)	10 (76.9%)	9 (81.8%)	49 (57%)
CpG	*60 (46.2%)*	*168 (56.8%)*	0 (0%)	6 (46.2%)	7 (63.6%)	43 (50%)
Homopolymer	27 (20.8%)	71 (24%)	1 (33.3%)	4 (30.8%)	2 (18.2%)	22 (25.6%)
	**GC- > AT**		**GC- > TA**		**GC- > CG**	
	**Control**	**BaP**	**Control**	**BaP**	**Control**	**BaP**
#Mutations	75	39	28	117	11	27
CpG	37 (49.3%)	20 (51.3%)	10 (35.7%)	**75 (64.1%)**	6 (54.5%)	**24 (88.9%)**
Homopolymer	14 (18.7%)	7 (17.9%)	5 (17.9%)	28 (23.9%)	4 (36.4%)	5 (18.5%)
	**AT- > GC**		**AT- > TA**		**AT- > CG**	
	**Control**	**BaP**	**Control**	**BaP**	**Control**	**BaP**
#Mutations	1	4	1	9	0	1
CpG	0 (0%)	0 (0%)	0 (0%)	0 (0%)	0	0 (0%)
Homopolymer	0 (0%)	1 (25%)	1 (100%)	4 (44.4%)	0	0 (0%)

**Bold** indicates p < 0.05 relative to controls.

Underline indicates p < 0.1.

**Table 3:** Analysis of sequence context of *lacZ* mutations identified in the bone marrow of male mice exposed to benzo(a)pyrene.

	**ALL MUTATIONS**		Insertion		Deletion	
	control	BaP	control	BaP	control	BaP
#Mutations	144	802	3	27	17	152
A:T	27 (18.8%)	108 (13.5%)	1 (33.3%)	8 (29.6%)	7 (41.2%)	62 (40.8%)
G:C	117 (81.2%)	694 (86.5%)	2 (66.7%)	19 (70.4%)	10 (58.8%)	90 (59.2%)
CpG	69 (47.9%)	**470 (58.6%)**	2 (66.7%)	8 (29.6%)	9 (52.9%)	81 (53.3%)
Homopoly	31 (21.5%)	156 (19.5%)	2 (66.7%)	9 (33.3%)	2 (11.8%)	34 (22.4%)
	**GC- > AT**		**GC- > TA**		**GC- > CG**	
	**Control**	**BaP**	**Control**	**BaP**	**Control**	**BaP**
#Mutations	45	111	47	345	13	129
CpG	24 (53.3%)	45 (40.5%)	27 (57.4%)	230 (66.7%)	7 (53.8%)	**106 (82.2%)**
Homopoly	13 (28.9%)	21 (18.9%)	7 (14.9%)	56 (16.2%)	2 (15.4%)	25 (19.4%)
	**AT- > GC**		**AT- > TA**		**AT- > CG**	
	**Control**	**BaP**	**Control**	**BaP**	**Control**	**BaP**
#Mutations	6	3	5	25	8	10
CpG	0 (0%)	0 (0%)	0 (0%)	0 (0%)	0 (0%)	0 (0%)
Homopoly	1 (16.7%)	0 (0%)	1 (20%)	6 (24%)	3 (37.5%)	5 (50%)

These data are derived from previously published results^16^.

**Bold** indicates p < 0.05 relative to controls.
