# Supplementary material for: Next generation sequencing of benzo(a)pyrene-induced lacZ mutants identifies a germ cell-specific mutation spectrum
Source: Sci Rep. 2016 Nov 10;6:36743. doi: 10.1038/srep36743 (PMC5103183; doi:10.1038/srep36743)

SUPPLEMENTARY INFORMATION

Next generation sequencing of benzo(a)pyrene-induced lacZ mutants identifies a germ cell-specific mutation spectrum

Jason M. O'Brien[1,2], Marc A. Beal[1], Carole L. Yauk[1] and Francesco Marchetti[1*]

[1]Environmental Health Science and Research Bureau, Health Canada, Ottawa, ON, Canada, K1A 0K9

[2]Present Address: Ecotoxicology and Wildlife Health Division, Environment Canada, Ottawa, ON, Canada, K1A 0H3

JMO'B: jason.obrien@canada.ca

MAB: marc.beal@canada.ca

CLY: carole.yauk@canada.ca

*Corresponding author:

FM: francesco.marchetti@canada.ca

Environmental Health Centre, Tunney's Pasture, 0803A, Ottawa, ON, Canada, K1A 0K9

Phone: (613) 957-3137

Fax: (613) 941-8530

Supplementary Figure 1: The frequency, by type, of spontaneous or BaP-induced *lacZ* mutations recovered from sperm 42 days after treatment. The frequency of each mutation type was determined by multiplying the *lacZ* mutation frequency after clonal correction (Table 1) by the proportion of each mutation type observed (Figure 2). For example, the mutation frequency for BaP after correction was 9.5×10^{-5}, and the proportion of G:C → T:A was 0.40 therefore, the mutation frequency of G:C → T:A in BaP was 3.8×10^{-5}.

Supplementary Table 1: The position, type, and clone count for each independent *lacZ* mutation sequenced.

Supplementary Table 2: Sequence context analysis of spontaneous or BaP-induced *lacZ* mutations recovered from sperm 42 days after treatment.

Supplementary Table 3: The position and frequency of spontaneous and BaP-induced mutations at identified mutation hotspots in *lacZ* transgenes recovered from sperm 42 days after treatment.

Supplementary Table 4: The proportion of spontaneous and BaP-induced base-substitutions that resulted in stop, missense or silent codon mutations in *lacZ* recovered from sperm 42 days after treatment.

Supplementary Figure 1

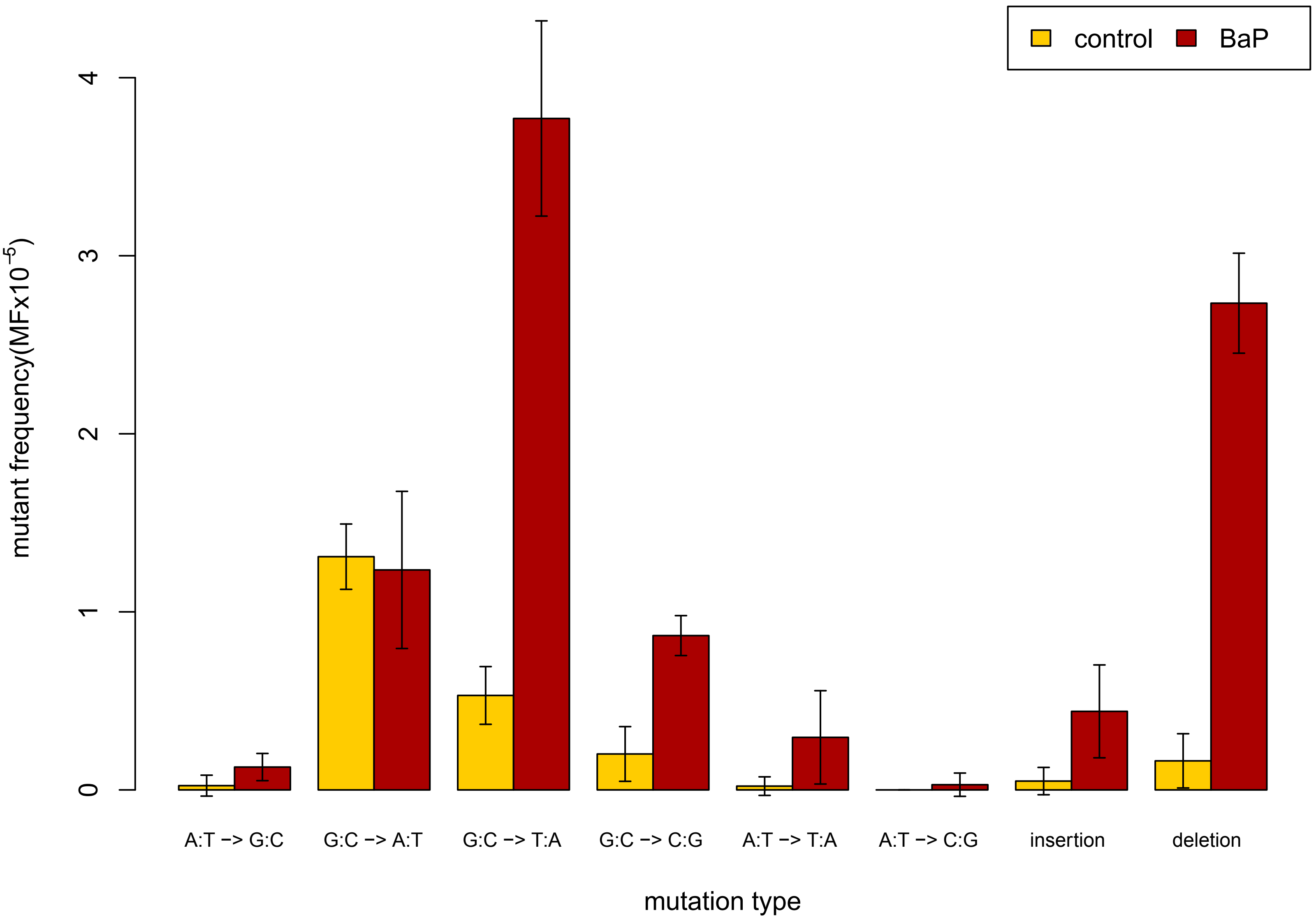

Supplementary Table 4: The proportion of spontaneous and BaP-induced base-substitutions that resulted in stop, missense or silent codon mutations in lacZ recovered from sperm 42 days after treatment.

	% Control	% BaP
stop	56.4	48.2
missense	43.6	46.2
silent	0	5.6

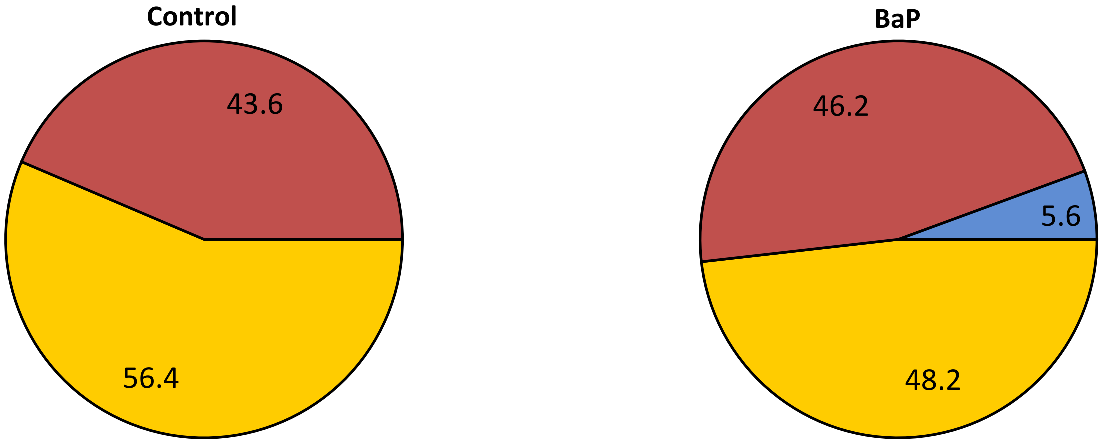

Supplement: Supplementary Information [file srep36743-s1.pdf]
